# RIFLE criteria for acute kidney injury are associated with hospital mortality in critically ill patients: a cohort analysis

**DOI:** 10.1186/cc4915

**Published:** 2006-05-12

**Authors:** Eric AJ Hoste, Gilles Clermont, Alexander Kersten, Ramesh Venkataraman, Derek C Angus, Dirk De Bacquer, John A Kellum

**Affiliations:** 1 The Clinical Research, Investigation, and Systems Modeling of Acute Illness (CRISMA) Laboratory, Department of Critical Care Medicine, University of Pittsburgh, School of Medicine, Pittsburgh, Pennsylvania, USA; 2 Intensive Care Unit, Ghent University Hospital, Gent, Belgium; 3 Department of Public Health, Ghent University, Gent, Belgium

## Abstract

**Introduction:**

The lack of a standard definition for acute kidney injury has resulted in a large variation in the reported incidence and associated mortality. RIFLE, a newly developed international consensus classification for acute kidney injury, defines three grades of severity – risk (class R), injury (class I) and failure (class F) – but has not yet been evaluated in a clinical series.

**Methods:**

We performed a retrospective cohort study, in seven intensive care units in a single tertiary care academic center, on 5,383 patients admitted during a one year period (1 July 2000–30 June 2001).

**Results:**

Acute kidney injury occurred in 67% of intensive care unit admissions, with maximum RIFLE class R, class I and class F in 12%, 27% and 28%, respectively. Of the 1,510 patients (28%) that reached a level of risk, 840 (56%) progressed. Patients with maximum RIFLE class R, class I and class F had hospital mortality rates of 8.8%, 11.4% and 26.3%, respectively, compared with 5.5% for patients without acute kidney injury. Additionally, acute kidney injury (hazard ratio, 1.7; 95% confidence interval, 1.28–2.13; *P *< 0.001) and maximum RIFLE class I (hazard ratio, 1.4; 95% confidence interval, 1.02–1.88; *P *= 0.037) and class F (hazard ratio, 2.7; 95% confidence interval, 2.03–3.55; *P *< 0.001) were associated with hospital mortality after adjusting for multiple covariates.

**Conclusion:**

In this general intensive care unit population, acute kidney 'risk, injury, failure', as defined by the newly developed RIFLE classification, is associated with increased hospital mortality and resource use. Patients with RIFLE class R are indeed at high risk of progression to class I or class F. Patients with RIFLE class I or class F incur a significantly increased length of stay and an increased risk of inhospital mortality compared with those who do not progress past class R or those who never develop acute kidney injury, even after adjusting for baseline severity of illness, case mix, race, gender and age.

## Introduction

Acute kidney injury is well recognized for its impact on the outcome of patients admitted to the intensive care unit (ICU). Illness severity scores such as the Acute Physiology and Chronic Health Evaluation version III (APACHE III) scoring system [1] and the Sequential Organ Failure Assessment score (SOFA) [2] both weight kidney dysfunction heavily (20% and 16.6% of the total scores for acute physiology). Yet there is no consensus on the amount of dysfunction that defines acute kidney injury, with more than 30 definitions in use in the literature today [3]. The variety of definitions used in clinical studies may be partly responsible for the large variations in the reported incidence (1–31%) [4-6] and the associated mortality (19–83%) [3,6-9] of acute kidney injury. Indeed, the lack of a uniform definition for acute kidney injury is believed to be a major impediment to research in the field [10]. Acute kidney injury is generally defined as 'an abrupt and sustained decrease in kidney function'. Until recently there has not been a consensus on how best to assess kidney function; namely, what markers best reflect kidney function, and what values of those markers discriminate normal from abnormal kidney function.

To establish a uniform definition for acute kidney injury, the Acute Dialysis Quality Initiative formulated the Risk, Injury, Failure, Loss, and End-stage Kidney (RIFLE) classification [11]. RIFLE defines three grades of increasing severity of acute kidney injury – risk (class R), injury (class I) and failure (class F) – and two outcome classes (loss and end-stage kidney disease) (see Table 1). A unique feature of the RIFLE classification is that it provides three grades of severity for acute kidney injury based on changes in either serum creatinine or urine output from the baseline condition. This allows classification of patients with acute kidney injury into one of the three RIFLE severity classes (Table 1).

RIFLE represents a new classification system issued from a process of formal evidence appraisal and expert opinion [11,12]. Three studies were recently published that used the RIFLE classification to evaluate the occurrence rate and/or outcome of acute kidney injury in two relatively small cohorts (207 ICU patients treated with renal replacement therapy and 183 ICU patients with acute kidney injury) and one larger cohort (813 patients after cardiac surgery) [13-15]. The clinical characteristics and predictive ability of this classification have not, however, been clinically validated in a large general ICU population. The aims of this study were therefore to characterize acute kidney injury defined by the maximum RIFLE classification, to examine the progression between stages of the classification, and to relate this classification to the length of stay and mortality in a large cohort of critically ill patients.

## Patients and methods

### Study population

We constructed a retrospective cohort of all adult hospitalizations during a 12 month period (1 July 2000–30 June 2001) at the University of Pittsburgh Medical Center that were admitted to one of its seven ICUs during their hospital stay. We excluded patients receiving chronic hemodialysis (*n *= 146) from the study cohort, and we only considered the first admission for patients who were readmitted to the ICU during the study period (*n *= 327). The University of Pittsburgh Medical Center is a tertiary care academic medical center with seven ICUs and more than 120 ICU beds serving medical, surgical, neurological, trauma and solid organ transplant patients.

### Data collection

The study was approved by the Institutional Review Board of the University of Pittsburgh Medical Center. Data from different sources were merged by a non-investigator data manager (such as, an honest broker) and were stripped of all identifying information to preserve patient anonymity and to comply with local and federal regulations. Demographic data were retrieved from the electronic hospital database, laboratory data were retrieved from the laboratory database, and patient data were retrieved from the electronic hospital records. After merging data from the different sources, we performed automated and manual data verification. The patient data included demographic, administrative, physiologic, laboratory and hospital outcome information. Ethnicity, reported as white, black or other, was reported by the admitting nurse, and was used to calculate the glomerular filtration rate assessed by the Modification of Diet in Renal Disease (MDRD) equation [16]. High-density (every two hours) physiologic data were only available while patients were in the ICU, while other data sources covered the entire hospitalization. Urine output was recorded at least once every two hours, and serum creatinine was measured at least once daily.

### RIFLE criteria

We classified patients according to the maximum RIFLE class (class R, class I or class F) reached during their hospital stay. The RIFLE class was determined based on the worst of either glomerular filtration rate criteria or urine output criteria. We used the change in serum creatinine level and urine output to classify patients according to the RIFLE criteria.

Patients who met any of the criteria of the RIFLE classification were classified as acute kidney injury patients. For patients without chronic kidney insufficiency as reported in the medical history, we calculated a serum creatinine level using the MDRD equation [16] (Cr_MDRD_) as recommended by the Acute Dialysis Quality Initiative, by solving the MDRD equation for serum creatinine assuming a glomerular filtration rate of 75 ml/minute/1.73 m^2^. We then used the lowest creatinine value among the hospital admission creatinine, the ICU admission creatinine or the Cr_MDRD _creatinine as the baseline value. Approximately one-half of patients were classified using the Cr_MDRD _as a baseline. None of these values differed by very much, however (mean difference between creatinine on admission and Cr_MDRD _= 0; interquartile range -0.3 to 0.3), and our results are not qualitatively different regardless of which baseline is used. For patients with a history of kidney insufficiency (but not on chronic dialysis) we used their hospital admission creatinine as their baseline. We did not evaluate the outcome classes of RIFLE (loss and end-stage kidney disease criteria) in this study.

### Severity of illness

The APACHE III [1] and the SOFA [2] scores were calculated based on the worst variables recorded during the first 24 hours of ICU admission. The nonrenal total SOFA score was calculated from the total SOFA score minus the points for kidney insufficiency. In addition, we calculated the SOFA score and the nonrenal SOFA score on basis of the worst variables recorded during the 24 hours preceding the maximum RIFLE class.

### Statistical analysis

The central tendency for continuous data is expressed as the mean ± standard deviation or the median (interquartile range). We tested continuous variables for normality by distribution plots. We compared means using the Student's *t *test when normally distributed, and the Mann-Whitney *U* test when not. Comparisons across multiple groups were performed using the F test, with Bonferroni correction for multiple comparisons [17]. When data were not normally distributed, we used the Kruskal-Wallis *H* analysis of variance test; significant changes over the observation period were tested with the Mann-Whitney *U *test.

We performed univariate and multivariable logistic regression to assess the impact of different baseline characteristics found to be significantly different over the four groups, on the occurrence of acute kidney injury and on maximum RIFLE class F. In the multivariable model, all covariates were entered simultaneously (enter method). We analyzed for collinearity by assessing correlation between covariates. For continuous variables we analyzed the relationship between the outcome and the variable with locally weighted scatterplot smoothing in order to assess whether categorization was necessary. Finally, the goodness of fit of the model was tested by means of the Hosmer-Lemeshow statistic.

We analyzed hospital survival across groups using the chi-square and the Kaplan-Meier methods, and we tested differences between groups using the log-rank test. Patients alive at hospital discharge were censored. We performed a Cox proportional hazards regression analysis to examine whether the maximum RIFLE class and the incidence of acute kidney injury (defined as patients who fulfilled one of the RIFLE classes) were associated with mortality.

To correct for differences in patient characteristics, we included simultaneously age, gender, race, the main reason for ICU admission, the medical or surgical admission category, and the nonrenal SOFA score on ICU admission or at the maximum RIFLE class in the model (enter method). The nonrenal SOFA score was chosen as a covariate to control for multicollinearity between the RIFLE classification and scoring systems that include points for kidney insufficiency such as the APACHE III and SOFA scores. Interactions between the 'main reason for admission' and the maximum RIFLE class were explored, and were found not to be significant.

We tested whether it was appropriate to treat continuous variables as continuous by a residuals plot. We tested the assumption of proportionality of hazards by plotting hazard rates against time for the four different categories, as well as by the numerical method proposed by Lin and colleagues [18] derived from cumulative sums of martingale residuals. We found no evidence of violating the proportional hazards assumption. Finally, we tested the qualitative goodness of fit of the model with residual plots. A double-sided *P *value less than 0.05 was considered significant. Analysis was performed with the statistical software package SPSS 11.0.1 (SPSS Inc., Chicago, IL, USA).

## Results

### Characteristics of patients with acute kidney injury

A total of 5,383 patients was evaluated. The baseline characteristics of the patient cohort are presented according to the maximum RIFLE class in Table 2. The four groups differed in age, race, pre-existing chronic kidney insufficiency, admission type, severity of illness on admission and at the time of maximum RIFLE class, APACHE III score, SOFA score and the nonrenal SOFA score, and the proportion of patients already admitted inhospital to another non-ICU ward.

Results of the regression analyses examining the impact of the different baseline characteristics on the appearance of acute kidney injury and maximum RIFLE class F are presented in Table 3. Increasing age, greater severity of illness (APACHE III, SOFA and nonrenal SOFA scores), pre-existing chronic kidney insufficiency and a preceding admission to a non-ICU ward in the hospital were associated with increased risk for occurrence of acute kidney injury and maximum RIFLE class F. In addition, black patients had increased risk for development of maximum RIFLE class F. Medical admissions were less likely to result in acute kidney injury or RIFLE class F compared with surgical admissions.

### Progression of acute kidney injury to maximum RIFLE class

**Figure 1:**
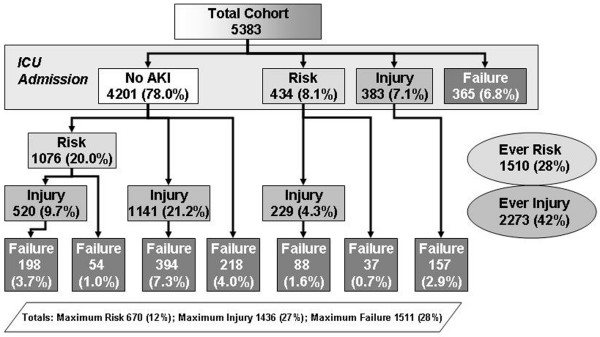
Flow chart of the clinical course of patients until the maximum RIFLE class. Data expressed as patient numbers who were identified at each level, and the percentage of the total number of patients. Patients who appear to skip a grade (class risk or class injury) do so because they did not remain at a transition state for at least 24 hours. 'Ever Risk' and 'Ever Failure' refers to the number of patients who could be identified at this stage. AKI, acute kidney injury; ICU, intensive care unit; RIFLE, Risk, Injury, Failure, Loss, and End-stage Kidney Disease.

The progression of acute kidney injury during the ICU stay to the maximum RIFLE class is shown in Figure 1. On the first day of ICU admission, 1,182 patients (21.9%) already had acute kidney injury, defined by the RIFLE criteria. During the entire ICU stay, 3,617 patients (67.2%) had an episode of acute kidney injury defined by RIFLE criteria. One-half of the patients reached the maximum RIFLE class within 2 days after ICU admission (Table 2).

More than 50% of the patients with RIFLE class R progressed to RIFLE class I or class F, and more than one-third of the patients with RIFLE class I progressed to class F. The time to progress to class I was 1 (0.5–3.6) days and the time to progress to class F was 4 (1.4–9.5) days. Patients who progressed to a higher RIFLE class during the ICU stay were older compared to patients whose renal function did not deteriorate, (62.4 ± 16.6 years versus 58.5 ± 18.0 years, *P *< 0.001) and were already more severely ill on admission, as illustrated by the greater APACHE III score (47 (36–62) versus 41 (29–56), *P *< 0.001) and the greater nonrenal SOFA score (5.7 (3.6) versus 4.8 (3.4), *P *< 0.001).

### Mortality, length of stay and renal replacement therapy

**Figure 2:**
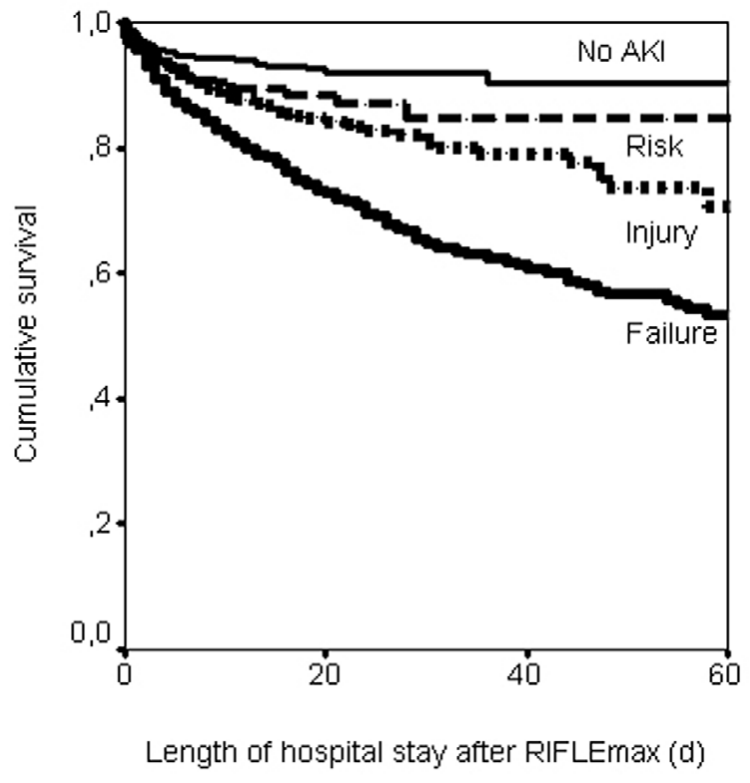
Kaplan-Meier curves for survival (inhospital) by maximum RIFLE class. Patients discharged alive were censored. Log-rank statistic, *P *< 0.001. AKI, acute kidney injury; RIFLE_max_, maximum Risk, Injury, Failure, Loss, and End-stage Kidney Disease (RIFLE) class during the intensive care unit stay (days).

Less than 1% of patients with maximum RIFLE class I and 14.2% of patients with maximum RIFLE class F received renal replacement therapy (Table 4). Increasing severity of acute kidney injury was associated with an increasing length of ICU stay and hospital stay, and higher mortality (Table 5 and Figure 2). Patients with maximum RIFLE class R, class I and class F had hospital mortality rates of 8.8%, 11.4% and 26.3%, respectively, compared with 5.5% for patients without acute kidney injury. Patients with maximum RIFLE class F based on glomerular filtration rate criteria had a somewhat higher inhospital mortality compared with patients who had a maximum RIFLE class F on urine output criteria (27.9% versus 21.9%, *P *= 0.020). The unadjusted hazard ratios (95% confidence interval) for hospital mortality for acute kidney injury and RIFLE class R, class I and class F were, respectively, 2.1 (1.67–2.57, *P *< 0.001), 1.3 (0.91–1.93, *P *= 0.142), 1.9 (1.45–2.48, *P *< 0.001) and 3.4 (2.64–4.29, *P *< 0.001). After adjustment for covariates, acute kidney injury was still associated with an almost twofold increased hazard for hospital mortality (Table 5, panel A). Maximum RIFLE class I and class F were both associated with mortality in the covariate-adjusted Cox regression model (Table 5, panel B). These results were unchanged when the nonrenal SOFA at the time of maximum RIFLE class was substituted for the nonrenal SOFA at ICU admission.

## Discussion

We found that acute kidney injury, defined by the RIFLE classification, had a high incidence (67.2%) and was associated with an increased risk for hospital mortality compared with those who never developed acute kidney injury. The incidence of almost 70% may appear at odds with the existing literature [5]. Even when limiting cases to those with RIFLE class F (28%) we found a higher rate for ICU patients than typically reported. Fourteen percent of class F patients received renal replacement therapy, however, leading to a rate of 4–5% among ICU patients, consistent with previous reports [9,19]. Indeed, our study highlights the potential for under-reporting when renal replacement therapy is used to 'define' acute kidney injury. Importantly, even milder degrees of kidney dysfunction, RIFLE class R or class I, were still associated with excess mortality compared with patients who maintained normal function. RIFLE provided a well-balanced classification system for determination of patients with different severity of acute kidney injury, at least as far as risk of mortality or need for renal replacement therapy is concerned.

Not surprisingly, the occurrence of acute kidney injury and maximum RIFLE class F were associated with increased baseline severity of illness and older age (Tables 2 and 3). Patients developing acute kidney injury were slightly older and had higher APACHE III and SOFA scores, even when kidney dysfunction was not counted. However, the severity within acute kidney injury was not so affected by these factors. Patients progressing to RIFLE class I and class F were no older and their nonrenal SOFA scores no greater than patients remaining in RIFLE class R. Although, Herget-Rosenthal and colleagues have also described the progression of acute kidney injury in a selected cohort of 85 ICU patients [20], this is to our knowledge the first time that the progression of acute kidney injury has been examined in a large dataset of general ICU patients.

RIFLE class R would appear to be aptly named. More than one-half of the patients of class R progressed to more severe RIFLE classes, yet those that did not were not at increased risk of hospital mortality. Future studies could target this population for prevention. RIFLE class I may also have been fortuitously named, for this is the stage at which risk for hospital mortality increases even after controlling for covariates. It was commonly held until fairly recently that patients die 'with, and not of, acute renal failure'. Medication (for example, erythropoietin and diuretics) and renal replacement therapy were thought to 'replace' the loss of kidney function. It has already been demonstrated in critically ill patients that severe acute renal failure, defined as the need for renal replacement therapy or oliguria, is independently associated with mortality [4,5,19,21]. In addition, in a cardiothoracic surgery population and in a cohort of hospitalized patients, both with a lower baseline mortality compared with general ICU patients, small changes in serum creatinine were associated with a worse outcome [22,23]. In the present study we confirm the association of acute kidney injury with increased hospital mortality in a general ICU population. This is a remarkable finding considering how common this condition appears to be – 55% of all patients had RIFLE class I or class F. Furthermore, there was increasing mortality risk over RIFLE classes, despite the fact that these ICU patients had similar comorbidity, as reflected by the nonrenal SOFA score.

The finding that moderate degrees of kidney dysfunction pose a significant risk of death is particularly notable given that we know very little of why this should be. Acute kidney injury may simply be colinear with unmeasured elements of comorbidity, or it may be causally related to the increased mortality. Future studies should consider exploring whether alternative management of patients with mild degrees of kidney dysfunction could change the outcome. If the problem is actually the kidney, then possible mechanisms underlying the excess mortality associated with acute kidney injury are likely to be found in the pathophysiologic changes resulting from kidney insufficiency and adverse effects of renal replacement therapy [24,25]. Salt and water retention resulting in volume overload, hyperkalemia and acid-base derangements [26], perhaps leading to decreased blood pressure, cardiac output, hepatic and renal blood flow [27], to insulin resistance and protein breakdown, and even to alterations in innate immunity [28], all may contribute to the excess mortality in this group of patients. Furthermore, patients with acute kidney injury have a high incidence of infectious complications [29-31] and frequently develop anemia. Finally, acute kidney injury itself can lead to a non-infectious, proinflammatory response with activation of leukocytes, secretion of proinflammatory cytokines and recruitment of neutrophils and macrophages with resultant lung injury, as has been demonstrated in animal models of ischemia-reperfusion-induced acute renal failure [32,33]. All these changes may occur prior to, or even in, patients never receiving renal replacement therapy. These same mechanisms, however, may explain why patients who are treated with a lower dose of renal replacement therapy have a worse survival [34-36].

Our study has certain limitations. First, we did not attempt to compare RIFLE with other classification systems; nor did we compare urine output and creatinine criteria, but rather used the criteria as proposed by the Acute Dialysis Quality Initiative workgroup, as the worst classification by each criterion. It is possible that urine output and creatinine criteria provide complementary information, which is lost when these criteria are combined.

The Acute Dialysis Quality Initiative recommended the use of a baseline serum creatinine, yet a true baseline is often unknown for patients admitted to the ICU. Several possible baseline values existed for our patients (hospital admission, ICU admission, or a calculated baseline from the MDRD equation). Our use of the lowest of these values for any given patient may have lead to a higher estimate of change and therefore a higher estimate of the incidence of acute kidney injury. Although the MDRD equation was developed and validated on a large number of patients, conflicting results have been published regarding the validation of this equation in different patient populations. We acknowledge that this equation is only a substitute for the actual glomerular filtration rate, but validation of this equation or developing an alternative for the MDRD-derived baseline creatinine was beyond the scope of this study.

We also acknowledge that some members of our research group have contributed to the consensus process by which RIFLE was developed and by which MDRD recommendations were made. In addition, patient follow-up in our study was limited to hospital discharge information.

Some patients may have died shortly after hospital discharge. As shown in Figure 2, the curves continue to separate, particularly for those in the class F group. Longer follow-up would also be required to examine the RIFLE endpoints 'loss' and 'end-stage disease'. Early renal replacement therapy may theoretically influence the criteria, and patients that would have reached class F could be classified in our study as class R or class I. Only four class I patients were treated with renal replacement therapy, however, and reclassification of these patients to class F does not influence our results.

Although our study is relatively large and included seven ICUs, it was conducted at a single medical center whose case mix and referral patterns may not be representative of other centers. The case mix of this study cohort could have hindered the detection of specific conditions that influence the development of acute kidney injury. Finally, our retrospective study design, using existing medical records, limited our ability to look outside the ICU and to collect information on potential mechanisms of injury. Our design also prohibited the use of more sophisticated measures of kidney function. Indeed, our assessment of time to progression of acute kidney injury may have been artificially lengthened due to daily measurement of creatinine – some patients appeared to skip class R or class I because of this limitation.

## Conclusion

In this general ICU population, acute kidney 'risk, injury, failure' as defined by the newly developed RIFLE classification is associated with increased hospital mortality and resource use. Patients with RIFLE class R are indeed at high risk of progression to class I or class F. Patients with RIFLE class I or class F incur a significantly increased length of stay and an increased risk of inhospital mortality compared with those who do not progress past class R or those who never develop acute kidney injury, even after adjusting for baseline severity of illness, case mix, race, gender and age.

## Key messages

• 	The RIFLE classification is a very sensitive definition of acute kidney injury: acute kidney injury defined by the RIFLE classification occurred in two thirds of general ICU patients.

• 	RIFLE classes injury and failure are independently associated with increased risk for in-hospital dead.

• 	Patients who meet the very sensitive RIFLE "risk" criteria, are at significant risk for progression to injury or failure, and therefore in-hospital dead.

## Abbreviations

APACHE III = Acute Physiology and Chronic Health Evaluation, version III; class F = failure, according to the RIFLE classification; class I = injury, according to the RIFLE classification; class R = risk, according to the RIFLE classification; Cr_MDRD _= serum creatinine based upon the MDRD equation; ICU = intensive care unit; MDRD = Modification of Diet in Renal Disease; RIFLE = Risk, Injury, Failure, Loss, and End-stage Kidney; SOFA = Sequential Organ Failure Assessment score; SOFA_nonrenal _= SOFA score without points for renal insufficiency.

## Competing interests

The authors declare that they have no competing interests.

## Authors' contributions

EAJH designed the study, analyzed the raw data set, performed the statistical analysis and contributed to writing of the paper. GC set up the raw data set, helped to design the study and contributed to writing of the paper. AK helped analyze the raw data set and helped in the design of the study. RV and DCA helped to design the study and contributed to writing the paper. DDB helped with the statistical analysis. JAK designed the study, analyzed the data and contributed to writing the paper. All authors read, edited and ultimately approved the final manuscript.

## Figures and Tables

**Table 1:** Risk, Injury, Failure, Loss, and End-stage Kidney (RIFLE) classification

Class	Glomerular filtration rate criteria	Urine output criteria
Risk	Serum creatinine × 1.5	< 0.5 ml/kg/hour × 6 hours
Injury	Serum creatinine × 2	< 0.5 ml/kg/hour × 12 hours
Failure	Serum creatinine × 3, or serum creatinine ≥ 4 mg/dl with an acute rise > 0.5 mg/dl	< 0.3 ml/kg/hour × 24 hours, or anuria × 12 hours
Loss	Persistent acute renal failure = complete loss of kidney function > 4 weeks	
End-stage kidney disease	End-stage kidney disease > 3 months	

For conversion of creatinine expressed in conventional units to SI units, multiply by 88.4. RIFLE class is determined based on the worst of either glomerular filtration criteria or urine output criteria. Glomerular filtration criteria are calculated as an increase of serum creatinine above the baseline serum creatinine level. Acute kidney injury should be both abrupt (within 1–7 days) and sustained (more than 24 hours). When the baseline serum creatinine is not known and patients are without a history of chronic kidney insufficiency, it is recommend to calculate a baseline serum creatinine using the Modification of Diet in Renal Disease equation for assessment of kidney function, assuming a glomerular filtration rate of 75 ml/min/1.73 m^2^. When the baseline serum creatinine is elevated, an abrupt rise of at least 0.5 mg/dl to more than 4 mg/dl is all that is required to achieve class Failure.

**Table 2:** Baseline characteristics of patients classified according to the maximum Risk, Injury, Failure, Loss, and End-stage Kidney (RIFLE) class

	No acute kidney injury	Risk	Injury	Failure
*n*	1,766 (32.8%)	670 (12.4%)	1,436 (26.7%)	1,511 (28.1%)
Sex (male)	988 (55.9%)	372 (55.5%)	841 (58.6%)	570 (57.0%)
Age (years)*	56.6 (18.2)	63.4 (17.0)	62.6 (16.6)	62.1 (16.4)
Race (*n *= 5,101)**				
White	1,491 (89.5%)	587 (91.3%)	1,237 (90.7%)	1,255 (87.9%)
Black	146 (8.8%)	45 (7.0%)	111 (8.1%)	156 (10.9%)
Other	29 (1.7%)	11 (1.7%)	16 (1.2%)	17 (1.2%)
Chronic kidney insufficiency*	17 (1.0%)	4 (0.6%)	17 (1.2%)	121 (8.0%)
Admission type* (*n *= 5,375)				
Medical	778 (44.2%)	277 (41.4%)	566 (39.4%)	545 (36.1%)
Surgical	984 (55.8%)	392 (58.6%)	870 (60.6%)	963 (63.9%)
Reason for admission according to organ system* (*n *= 5,375)				
Cardiovascular disease	446 (25.3%)	236 (35.3%)	446 (31.1%)	513 (34.0%)
Neurological disease	311 (17.7%)	109 (16.3%)	314 (21.9%)	327 (21.7%)
Pulmonary disease/infection	226 (12.8%)	101 (15.1%)	232 (16.2%)	291 (19.3%)
Trauma	317 (18.0%)	85 (12.7%)	203 (14.1%)	163 (10.8%)
Malignancy	117 (6.6%)	34 (5.1%)	38 (2.6%)	35 (2.3%)
Gastrointestinal disease	67 (3.8%)	22 (3.3%)	32 (2.2%)	26 (1.7%)
Other	278 (15.8%)	82 (12.3%)	171 (11.9%)	153 (10.1%)
APACHE III score* (*n *= 3,400)	36 (26–47)	46 (35–57)	46 (36–58)	56 (41–73)
SOFA score*	5.1 (3.3)	6.3 (4.0)	6.8 (4.1)	7.8 (4.5)
SOFA_nonrenal _score*	4.5 (3.1)	5.3 (3.5)	5.6 (3.5)	5.9 (3.9)
SOFA RIFLE_max _score* (*n *= 4,994)	3.7 (3.2)	5.3 (3.8)	5.9 (3.8)	6.7 (4.3)
SOFA_nonrenal _RIFLE_max _score* (*n *= 4,994)	3.2 (2.8)	4.5 (3.5)	5.0 (3.4)	5.0 (3.7)
Inhospital before ICU admission*	527 (29.8%)	243 (36.3%)	476 (33.1%)	592 (39.2%)
Pre-ICU LOS* (days)	1 (1–4)	2 (1–4)	2 (1–5)	2 (1–6)
Time to RIFLE_max_* (days)		2 (1–3)	2 (1–4)	2 (1–7)
RIFLE class on glomerular filtration rate criteria*		463 (69.1%)	929 (64.7%)	1,110 (73.5%)

Continuous variables are presented as the mean (standard deviation) when normally distributed or as the median (interquartile interval) when not normally distributed. Categorical variables are presented as percentages. No acute kidney injury is those patients without any occurrence of RIFLE criteria; APACHE III, Acute Physiology and Chronic Health Evaluation, version III; SOFA_nonrenal_, Sequential Organ Failure Assessment score without points for renal insufficiency, SOFA RIFLE_max_, Sequential Organ Failure Assessment score at the time of maximum RIFLE class; pre-ICU LOS, length of hospital stay before intensive care unit admission (only for patients who were in hospital before intensive care unit admission). *P < 0.001, **P = 0.035.

**Table 3:** Impact of baseline characteristics on the occurrence of acute kidney injury (multivariate logistic regression analysis)

Characteristic	Covariates associated with occurrence of acute kidney injury		Covariates associated with occurrence of maximum RIFLE class failure	
	
	Odds ratio (95% confidence interval)	*P*	Odds ratio (95% confidence interval)	*P*
Age (per year older)	1.02 (1.02–1.03)	< 0.001	1.01 (1.00–1.01)	0.001
Race (reference white)		0.130		0.001
Black	1.20 (0.96–1.50)	0.111	1.50 (1.21–1.86)	< 0.001
Other	0.73 (0.44–1.23)	0.237	0.78 (0.41–1.38)	0.397
Chronic kidney insufficiency	4.19 (2.48–7.10)	< 0.001	8.86 (6.01–13.05)	< 0.001
Medical admission (reference surgical)	0.79 (0.69–0.90)	< 0.001	0.76 (0.66–0.87)	< 0.001
Reason for admission according to organ system (reference cardiovascular disease)		< 0.001		< 0.001
Trauma	0.64 (0.53–0.79)	< 0.001	0.64 (0.52–0.80)	< 0.001
Neurological disease	0.93 (0.78–1.13)	0.481	1.02 (0.85–1.2)	0.830
Pulmonary disease and infection	1.08 (0.88–1.32)	0.461	1.16 (0.96–1.40)	0.120
Gastrointestinal disease	0.51 (0.35–0.73)	< 0.001	0.51 (0.32–0.66)	0.004
Malignancy	0.36 (0.27–0.49)	< 0.001	0.45 (0.31–0.66)	< 0.001
Other	0.57 (0.47–0.70)	< 0.001	0.60 (0.48–0.74)	< 0.001
SOFA_nonrenal _(per point greater)	1.19 (1.16–1.21)	< 0.001	1.08 (1.06–1.10)	< 0.001
In hospital before ICU admission	1.18 (1.03–1.36)	0.015	1.19 (1.04–1.36)	0.012

SOFA_nonrenal_, Sequential Organ Failure Assessment score without points for kidney insufficiency; ICU, intensive care unit. The odds ratios were calculated with logistic regression analysis. The goodness of fit of the multivariable regression model was tested by the Hosmer-Lemeshow statistic: *P *= 0.080 for the model with acute kidney injury as the endpoint, and *P *= 0.019 for the model with maximum Risk, Injury, Failure, Loss, and End-stage Kidney (RIFLE) class failure as the endpoint.

**Table 4:** Outcomes for all patients and for patients classified according to the maximum Risk, Injury, Failure, Loss, and End-stage Kidney (RIFLE) class

	No acute kidney injury (*n *= 1,766)	Risk (*n *= 670)	Injury (*n *= 1,436)	Failure (*n *= 1,511)	All injury (*n *= 5,383)
Renal replacement therapy*	1 (0.1%)	0 (0%)	4 (0.3%)	214 (14.2%)	219 (4.1%)
Hospital LOS after reaching maximum RIFLE class (days)*	5 (3–10)	5 (3–10)	7 (4–14)	11 (5–23)	7 (3–14)
ICU LOS (days)*	3 (2–4)	3 (2–6)	5 (3–10)	9 (4–21)	4 (2–9)
Hospital LOS (days)*	6 (4–10)	8 (5–14)	10 (6–19)	16 (9–31)	9 (5–19)
Hospital mortality*	97 (5.5%)	59 (8.8%)	163 (11.4%)	398 (26.3%)	717 (13.3%)

Continuous variables presented as the median (interquartile interval) and categorical variables presented as the percentage. LOS, length of stay; ICU, intensive care unit. *P = 0.001 between the four subgroups no acute kidney injury, RIFLE class risk, RIFLE class injury and RIFLE class failure.

**Table 5:** Association of Risk, Injury, Failure, Loss, and End-stage Kidney (RIFLE) criteria with mortality

	Hazard ratio (95% confidence interval)	*P*
**Panel A: association of AKI, defined by meeting any RIFLE criteria, with mortality**		
AKI (reference no AKI)	1.7 (1.28–2.13)	< 0.001
SOFA_nonrenal _score (per point)	1.1 (1.10–1.15)	< 0.001
Age (per 10 years)	1.1 (1.07–1.18)	< 0.001
Sex (female)	0.9 (0.78–1.07)	0.252
Race (reference white)		0.528
Black	1.1 (0.88–1.45)	0.324
Other	0.8 (0.41–1.68)	0.608
Medical admission (reference surgical admission)	2.9 (2.44–3.40)	< 0.001
Main reason for admission (reference cardiovascular disease)		< 0.001
Trauma	0.9 (0.66–1.09)	0.196
Neurological disease	1.3 (1.03–1.52)	0.026
Pulmonary disease, infection	0.8 (0.65–1.03)	0.087
Gastrointestinal disease	0.5 (0.21–1.25)	0.140
Malignancy	0.2 (0.04–0.63)	0.009
Other	0.4 (0.22–0.62)	< 0.001
**Panel B: association of maximum RIFLE class with mortality**		
RIFLE_max _(reference no AKI)		< 0.001
Risk	1.0 (0.68–1.56)	0.896
Injury	1.4 (1.02–1.88)	0.037
Failure	2.7 (2.03–3.55)	< 0.001
SOFA_nonrenal _score (per point)	1.1 (1.10–1.14)	< 0.001
Age (per 10 years)	1.1 (1.08–1.20)	< 0.001
Sex (female)	0.9 (0.76–1.06)	0.190
Race (reference white)		0.673
Black	1.1 (0.87–1.45)	0.389
Other	0.9 (0.44–1.97)	0.844
Medical admission (reference surgical admission)	2.7 (2.27–3.21)	< 0.001
Main reason for admission (reference cardiovascular disease)		< 0.001
Trauma	0.9 (0.69–1.18)	0.463
Neurological disease	1.3 (1.06–1.60)	0.011
Pulmonary disease, infection	0.8 (0.66–1.07)	0.154
Gastrointestinal disease	0.6 (0.24–1.41)	0.230
Malignancy	0.2 (0.04–0.69)	0.013
Other	0.3 (0.20–0.60	< 0.001

Covariate-adjusted Cox proportional hazard regression analysis. AKI, acute kidney injury, patients meeting at least one of the RIFLE criteria; RIFLE_max_, maximum RIFLE class; SOFA_nonrenal_, Sequential Organ Failure Assessment score without points for kidney failure, determined on data from the first 24 hours of admission.
